# Sialolithotomy of the submandibular duct using sialendoscopy

**DOI:** 10.1186/s40902-019-0207-3

**Published:** 2019-06-24

**Authors:** Dong-Keon Lee, Euy-Hyun Kim, Chang-Woo Kim, Mong-Hun Kang, In-Seok Song, Sang-Ho Jun

**Affiliations:** Department of Oral and Maxillofacial Surgery, Korea University Anam Hospital, 73, Inchon-ro, Seongbuk-gu, Seoul, Republic of Korea

**Keywords:** Sialendoscopy, Sialolithotomy, Submandibular gland, Sialocentesis, Hyposalivation, Sialadenitis

## Abstract

**Background:**

Conventionally, indirect radiography has been used to diagnose salivary gland diseases. However, with the development of sialendoscopy, diagnosis and treatment of salivary gland diseases have become more effective. Herein, we report a case of sialolithotomy treated with sialendoscopy and compare it with the existing methods through a literature review.

**Case presentation:**

Two patients with a foreign body sensation under the tongue and dry mouth visited the Anam Hospital, Korea University. Radiographic examination revealed salivary stones inside the right Wharton duct, and the patients underwent sialolithotomy under local or general anaesthesia. The stones were totally removed, and there were no postoperative complications such as bleeding or pain.

**Conclusion:**

The development of sialendoscopy has enabled better definitive diagnosis of salivary gland diseases compared with the conventional methods; better treatment outcomes can be obtained when sialendoscopy is used in appropriate cases.

## Background

Chronic obstructive salivary gland disease can be caused by salivary stones, mucus plugs, duct stenosis, foreign bodies, or anatomical variation in the ductal system of salivary glands, and it can lead to the retention of saliva in the duct, discomfort, or infection, if not treated properly [1, 2].

In particular, sialolithiasis (also termed salivary calculi or salivary stones) is a condition in which a calcified mass forms within a salivary gland, usually in the duct of the submandibular gland (also termed ‘Wharton’s duct’), due to anatomical and physiological factors. It can cause swelling and pain in the affected salivary gland and worsens when salivary flow is stimulated. If it does not get removed spontaneously, the condition is usually managed by removing the stone surgically [2–4].

Previously, radiosialography, ultrasound, and magnetic resonance sialography have been used for the diagnosis of these salivary gland diseases. Radiosialography is the commonly used imaging technique for the study of salivary gland diseases, especially in the case of calculi [4, 5]; however, it is contraindicated in acute infection or in case of sensitivity to contrast medium. Ultrasonography is a first-level, noninvasive, and inexpensive imaging technique for the study of salivary gland disease without contrast medium. However, the sensitivity and specificity of the results vary widely because less mineralised or early-stage stones may not be detected, and they depend on the experience and expertise of the examiner. Other diagnostic methods that also aid in visualising salivary gland diseases indirectly, such as computed tomography (CT), magnetic resonance imaging (MRI), MR sialography, or scintigraphy, are expensive and need additional procedural steps with no specific advantage [4, 6–8].

At present, with the development of sialendoscopy, a noninvasive approach has emerged, and it has been effectively used to establish the diagnosis and treatment plan for the diseases of salivary duct system and glands. Sialendoscopy allows direct access into the duct, dilatation and irrigation of the duct system, and confirmation of its patency with a minimally invasive approach that widens or bypasses the stenosis [1, 4].

In this study report, we discuss the cases of two patients who underwent sialolithotomy with a sialendoscope at Korea University Anam Hospital. Additionally, we will discuss the differences from conventional techniques.

## Case presentation

### Case 1

#### Patient information

**Fig. 1:**
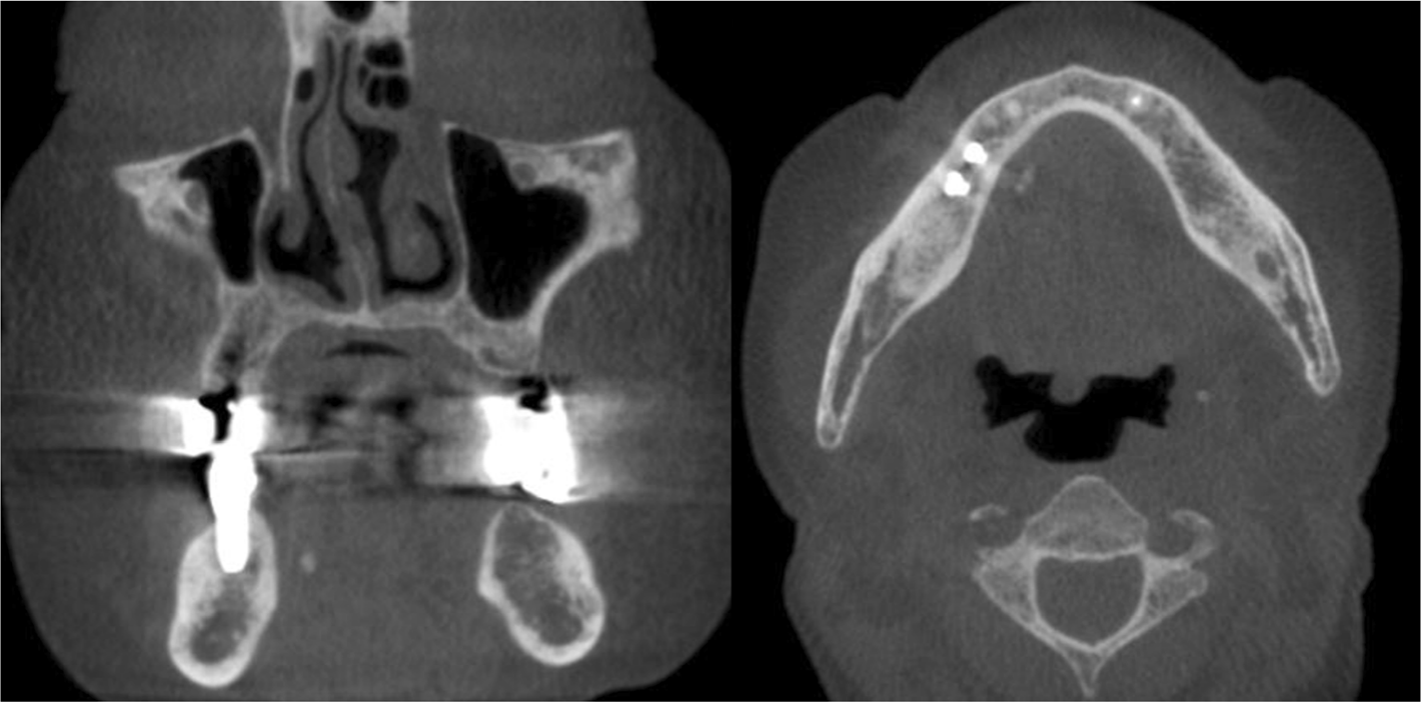
Salivary stones measuring 2 × 10 mm are detected in the right submandibular gland with cone-beam CT

An 81-year-old female patient visited the dental clinic with a foreign body sensation under the tongue and dry mouth on February, 2018. Papillary thyroid carcinoma was diagnosed, and a total thyroidectomy was performed in June 2003. Other underlying diseases were hypertension and chronic kidney disease. A sialolith was found in the right mandibular submandibular duct in the examination, and a sialolithotomy with sialendoscope was planned (Fig. 1). Preoperative and postoperative clinical symptoms of the surgical site were recorded.

#### Surgical procedure

**Fig. 2:**
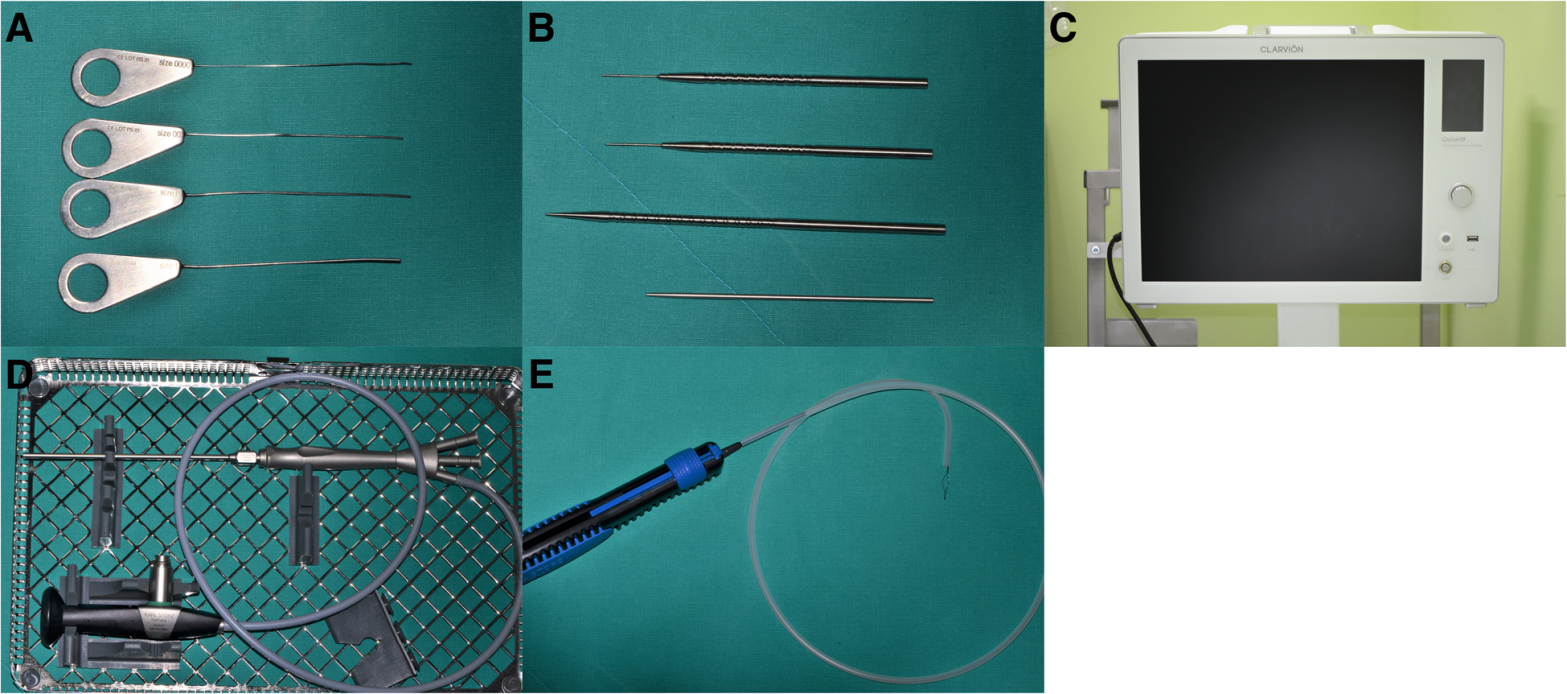
Sialendoscopic appliances. **a** Duct probe. **b** Orifice dilator. **c** Endoscopic monitor. **d** Three-port endoscope. **e** Three-wire basket

**Fig. 3:**
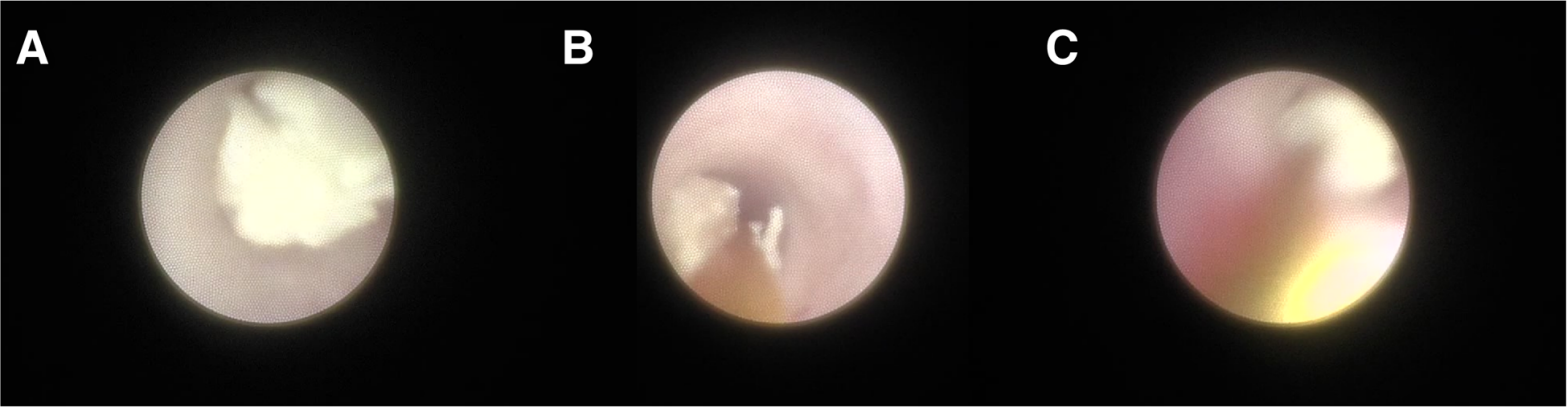
Endoscopic view during sialolithotomy. **a** Stones in the duct are observed. **b** Basket is approaching to engage stones. **c** Stones are engaging in the basket and removed from the duct

**Fig. 4:**
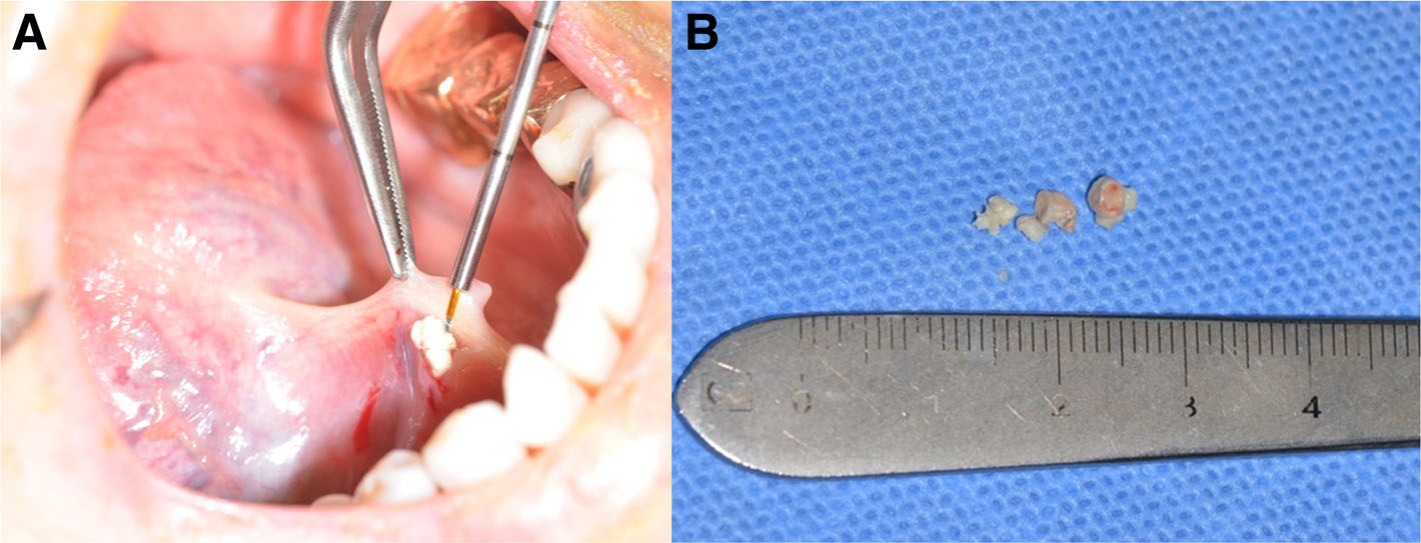
**a** The stones are coming out through the orifice of the submandibular duct. **b** Removed salivary stones

Extraoral and intraoral disinfection was done with a betadine-soaked cotton ball. After disinfection, the oral cavity was thoroughly washed with sterile saline. Local anaesthesia was performed on the tongue and mouth floor with 2% 1:100,000 lidocaine HCl-epinephrine, and the tongue was sutured using 3-0 silk to elevate it and secure the field of view of the surgical site. Through a microscope, the entrance of the right submandibular gland’s duct was confirmed, and a #0000 probe was carefully and sequentially inserted (Fig. 2) along the duct and expanded to a #3 probe. To relieve the stenosis of the duct orifice and allow access to the endoscope inside the duct, the orifice was expanded using a dilator. Sialendoscopy was done under saline irrigation, and the stone was confirmed. Using a three-wire basket, the stone was removed from the duct (Fig. 3). The size of the ductal orifice was smaller than that of the stone; a 0.1-cm incision was made at the entrance of the duct. After removal of the stone, the endoscope was reinserted to confirm that all the in situ stones were removed (Fig. 4). Then, the duct system was washed with a steroid solution. There was no postoperative bleeding or abnormality such as oedema and pain. Daily dressing and clinical examination to check for complications, such as infection, was performed; there were no unusual side effects after the operation.

### Case 2

#### Patient information

**Fig. 5:**
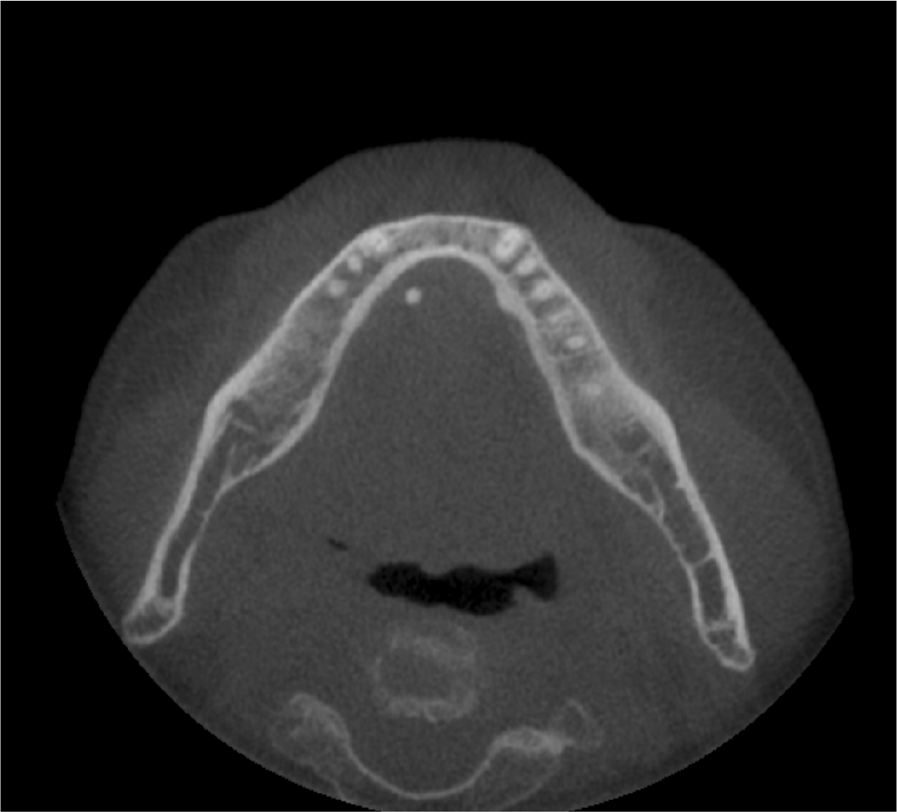
Salivary stones measuring 3 × 4 mm are detected in the right submandibular gland with cone-beam CT

A 66-year-old man visited the dental clinic because of the blockage of salivary duct of the right mandibular submandibular gland (Fig. 5) on June, 2018. From 7 to 8 years ago, there was a swelling and pain in the lower neck while eating food. Sialolithotomy was performed in other hospitals 4 to 5 years ago, but failed because of unstable movement of the stone during surgery. There was no systemic abnormality.

#### Surgical procedure

**Fig. 6:**
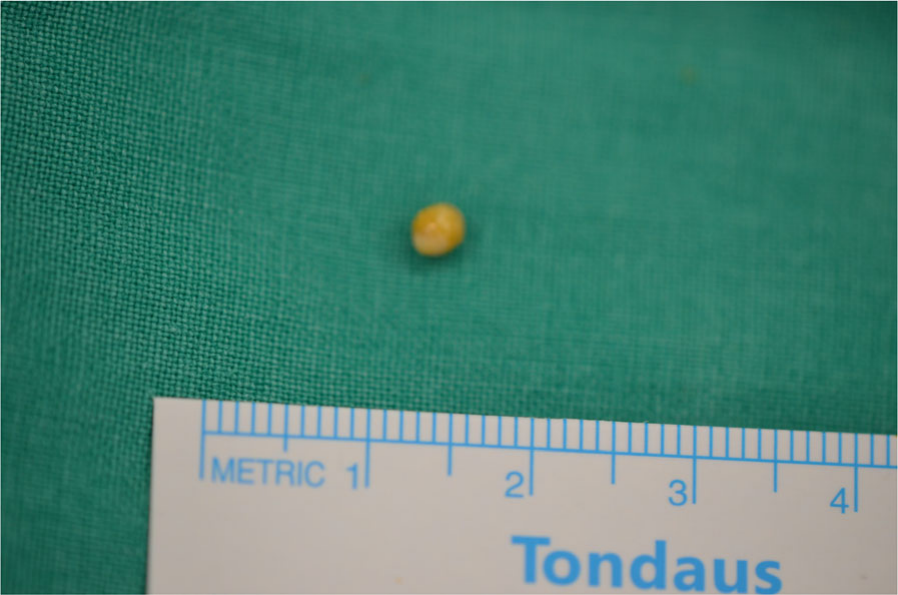
Removed stone

Extraoral and intraoral disinfection was done with a betadine-soaked cotton ball. After disinfection, the oral cavity was thoroughly washed with sterile saline. The patient underwent general anaesthesia through a nasotracheal intubation, and the tongue was sutured using 3-0 silk. The operation procedure was the same as the previous case (Fig. 6). There was no postoperative bleeding or abnormality such as oedema and pain. Daily dressing and clinical examination to check for complications, such as infection, was performed; the patient was discharged the next day without any discomfort.

## Discussion

The symptoms of obstructive salivary gland diseases include recurrent, painful swelling of the major salivary glands, and these symptoms can lead to a marked reduction in the patient’s quality of life [3, 9]. Previously, radiosialography, sonography, and MR sialography were used to diagnose salivary gland diseases. Radiosialography is the primary method of examination of salivary ducts, which can be used to diagnose gland lesions by acquiring images after injecting contrast media into the duct. However, it is contraindicated when there is an adverse reaction to the contrast media or an acute infection. Sonography is noninvasive and useful for the diagnosis of salivary gland calculi, but this technique depends on the experience and technique of the surgeon and is difficult to use for diagnosing disorders in the duct. MR sialography is another noninvasive method that can overcome the existing limitation by showing the entire salivary duct system, but it can cause distortion artifacts due to intraoral restorations. However, as the introduction of salivary endoscopy enabled direct visualisation of the duct system, these diagnostic limitations were overcome. Moreover, it is less invasive than conventional surgical methods for patients with stenosis or stones in the duct system or sialadenitis, which can reduce the discomfort of patients and alleviate symptoms [1, 4, 6, 10].

A few research groups have also investigated patient satisfaction after sialendoscopy-guided treatment for obstructive salivary gland diseases. Kroll et al., using the Short Form 36 (SF-36) questionnaire, found a high level of patient satisfaction [9, 11]. Aubin-Pouliot et al., using a questionnaire designed to obtain a chronic obstructive sialadenitis score showed similar results. The results showed that symptoms decreased significantly after sialendoscopy-assisted salivary duct surgery in submandibular glands more than in parotid glands [9, 12].

Recent studies have shown that microsialoliths play a major role in the pathogenesis of chronic sialadenitis. They can accumulate in normal salivary glands and lead to obstructive atrophy. This atrophy enables colonisation and proliferation of microbes, causing inflammation in the periphery duct system, followed by more severe atrophy and progressive infection, leading to chronic sialadenitis [3]. According to Quinn et al., intraductal instillation of penicillin allows antibiotics to reach the remaining microbes in the parenchyma, thus helping to relieve symptoms, but equally good results can be obtained with saline irrigation itself, which may be considered a more important factor [3, 13].

Radioactive iodine (RAI) therapy is another cause of salivary gland disease. According to Kim et al., chronic sialadenitis is the most common complication of RAI for the removal of remnant tissue after thyroidectomy. The prevalence of chronic RAI sialadenitis is 11–65% after RAI therapy. RAI-induced salivary gland damage results in obstructive sialadenitis, presenting recurrent swelling with or without pain at mealtime. In the chronic state, it further causes hypo-salivation and leads to other complications, such as speech and swallowing difficulties, taste alterations, oral candidiasis, and dental caries. Currently, RAI-induced chronic sialadenitis is treated conservatively by ensuring good oral hygiene and frequent hydration, using salivary substitutes and salivary stimulatory treatments, such as gland massage [14, 15]..

According to Kim’s study, sialendoscopy showing sialocentesis effect (intraductal irrigation with sterilized normal saline) could improve obstructive symptoms 3 months after sialendoscopic mechanical dilation; however, sialendoscopic treatment for chronic RAI sialadenitis was found to have some limitations in its ability to relieve xerostomia and improve weakened salivary gland functions [15].

This study was conducted to report the use of a sialendoscope in removing salivary stone. Recently, the development of salivary endoscopy has overcome the limitations of conventional methods and has become applicable in the diagnosis and treatment of salivary stones. Further, the patients’ discomfort is less compared with the conventional procedures. In this case, the patient had complete relief from foreign body sensation after surgery and showed slight relief from dryness. There were no significant complications after the operation and no significant side effects during the follow-up period.

## Conclusion

We reported two cases of noninvasive sialolithotomy with sialendoscopy instead of the conventional methods. If a sialendoscope is applied in the appropriate case not only in sialolithiasis but also in duct stenosis and sialadenitis, the patient will be less likely to experience discomfort and complications and can expect good treatment outcomes. For determining the long-term effects, further study is needed with a larger number of patients and longer follow-up. This research did not receive any specific grant from funding agencies in the public, commercial, or not-for-profit sectors.

## Data Availability

Please contact author for data requests.

## Abbreviations

CT: Computed tomography
HCl: Hydrogen chloride
MR: Magnetic resonance
RAI: Radioactive iodine
